# Neural-immune-cardiovascular axis: from mechanistic crosstalk to therapeutic targets in cardiovascular disease

**DOI:** 10.3389/fimmu.2026.1886102

**Published:** 2026-07-22

**Authors:** Junkang Cheng, Shuang Gao, Haowei Zhang, Wei Gao, Zeyuan Mei, Xiaoling Liu, Jocelyn Gao, Chenghu Guo, Guipeng An

**Affiliations:** 1 State Key Laboratory for Innovation and Transformation of Luobing Theory; Key Laboratory of Cardiovascular Remodeling and Function Research of Ministry of Education (MOE), National Health Commission (NCH), Chinese Academy of Medical Sciences (CAMS) and Shandong Province; Department of Cardiology, Qilu Hospital of Shandong University, Jinan, China; 2 Department of Cardiology, Jinan First People’s Hospital, Jinan, China; 3 The Sidney Kimmel Medical College at Thomas Jefferson University, Philadelphia, PA, United States

**Keywords:** atherosclerosis, autonomic nervous system, cardiovascular diseases, inflammation, neural-immune-cardiovascular axis, neuroimmunity, therapeutic targets

## Abstract

The neural-immune-cardiovascular axis represents an emerging and highly integrated physiological and pathophysiological concept, describing a complex bidirectional communication network between the nervous, immune, and vascular systems. This review systematically examines the pivotal role of this axis in maintaining cardiovascular homeostasis and in the pathogenesis of cardiovascular diseases. We first provide an overview of the fundamental signaling pathways between the components of this axis. Subsequently, we delve into the specific crosstalk mechanisms within the axis in the context of major cardiovascular conditions, including atherosclerosis, hypertension, and heart failure. A central focus is placed on critically evaluating the potential therapeutic targets and intervention strategies that have emerged from our understanding of this axis’s mechanisms. This review aims to provide a comprehensive perspective for understanding the multi-system pathogenesis of cardiovascular diseases and for informing the development of novel therapeutic approaches.

## 1 Introduction

The human heart, long perceived as a mere mechanical pump, can be profoundly and acutely broken by the mind. This is vividly exemplified by Takotsubo syndrome (TTS), often termed “broken heart syndrome,” a transient form of acute heart failure typically triggered by severe emotional or psychological stress, such as the grief of loss, intense fear, or overwhelming anxiety (1). The condition predominantly affects postmenopausal women and presents with symptoms mimicking an acute coronary syndrome, including chest pain and electrocardiographic changes, but in the absence of obstructive coronary artery disease (2). The COVID-19 pandemic provided a stark, real-world illustration of this mind-heart connection, where the profound psychological stressors of social isolation, fear of infection, and societal upheaval led to a documented surge in TTS cases, even among individuals without evidence of SARS-CoV-2 infection itself (3). This phenomenon underscores that emotional distress can be directly transduced into myocardial injury, challenging the traditional cardiocentric view of heart disease. The pathophysiology of TTS is thought to involve a catecholamine storm, where a massive sympathetic nervous system (SNS) outflow in response to stress leads to excessive epinephrine and norepinephrine release, causing direct myocardial stunning and microvascular dysfunction (1). This clinical narrative serves as a compelling entry point into a far more extensive and intricate biological dialogue, revealing that the heart does not operate in isolation but is a central participant in a continuous, multidirectional conversation orchestrated by the brain and mediated by the immune system.

This narrative challenges the conventional paradigm of cardiovascular disease (CVD) research, which has historically focused on local vascular pathology, lipid metabolism, and hemodynamic forces, often overlooking the brain as the central controller and the immune system as a critical effector. CVD remains the leading cause of global mortality, and while traditional risk factors like hypertension and dyslipidemia are well-established, they do not fully account for disease susceptibility or progression. Accumulating evidence now compellingly demonstrates that the nervous and immune systems are indispensable actors in the initiation, progression, and complications of CVD (4). This recognition has given rise to the integrative concept of the “neuroimmune cardiovascular axis” (or neuroimmune-cardiovascular axis) (5). This axis represents a dynamic, bidirectional communication network where the central nervous system (CNS), through the autonomic nervous system (ANS) and neuroendocrine pathways, engages in real-time dialogue with the peripheral immune system and the cardiovascular system itself (6). It is not a simple linear pathway but a complex circuit involving afferent sensory nerves that relay signals from the heart and vessels to the brain, and efferent sympathetic and parasympathetic nerves that convey commands back to cardiovascular and immune tissues (7). This communication is facilitated by a rich language of mediators, including neurotransmitters (e.g., norepinephrine, acetylcholine), neuropeptides (e.g., neuropeptide Y, substance P), cytokines, chemokines, and hormones, which collectively fine-tune vascular tone, immune surveillance, and tissue repair under physiological conditions (8).

In pathological states, however, this finely tuned axis becomes dysregulated, driving cardiovascular pathogenesis. Chronic psychosocial stressors, such as social isolation or loneliness, can lead to sustained activation of the SNS and the hypothalamic-pituitary-adrenal (HPA) axis, creating a state of chronic low-grade inflammation and oxidative stress that accelerates atherosclerosis (9). This neuroimmune dysregulation is a central mechanism in hypertension, where immune cell infiltration into the vessel wall, kidney, and key cardiovascular regulatory centers in the brain contributes to increased vascular resistance and sympathetic tone (10). For instance, activated T cells and macrophages release pro-inflammatory cytokines like interleukin-6 (IL-6), interleukin-17 (IL-17), and tumor necrosis factor-alpha (TNF-α), which promote endothelial dysfunction, oxidative stress, and vascular remodeling (11). Conversely, signals from diseased cardiovascular tissues feed back to the CNS. In atherosclerosis, specialized “neuroimmune cardiovascular interfaces” (NICIs) form in the adventitia of diseased arteries, where expanded axon networks interact closely with immune cells (12). These NICIs are part of a structural “artery-brain circuit” (ABC), where nociceptive afferents from the arterial adventitia project to the spinal cord and higher brain regions like the amygdala, while sympathetic efferents from the brainstem and hypothalamus project back to the adventitia via ganglia (12). Activation of this circuit, including increased splenic sympathetic nerve activity, can exacerbate disease progression, while its therapeutic disruption attenuates atherosclerosis (12). Similarly, after stroke, neuroinflammation driven by activated microglia disrupts cardiovascular-related neural networks and the blood-brain barrier, allowing central immune components to influence peripheral immunity, leading to splenic activation and subsequent myocardial infiltration by monocytes, causing cardiovascular dysfunction (13). This evidence firmly establishes that the neuroimmune cardiovascular axis is a fundamental regulatory layer whose disruption is a critical driver of major cardiovascular conditions, from hypertension and atherosclerosis to myocardial infarction and heart failure.

## 2 Core components and fundamental communication mechanisms of the neural-immune-caridiovascular axis

### 2.1 Neural and neuroendocrine output pathways

The nervous system exerts profound control over cardiovascular and immune functions through its efferent outputs, primarily mediated by the autonomic and sensory nervous systems. The sympathetic nervous system (SNS) plays a dominant role in this regulation. Sympathetic nerve terminals release norepinephrine (NE), which acts on specific adrenergic receptor subtypes expressed on distinct cell populations. On macrophages, NE primarily engages β2-adrenergic receptors (β2-AR) to modulate cytokine production—suppressing TNF-α and IL-12 while enhancing IL-10 at low concentrations, but paradoxically promoting pro-inflammatory cytokine release under conditions of chronic sympathetic overstimulation. On vascular smooth muscle cells (VSMCs), NE activates α1-adrenergic receptors (α1-AR) to drive vasoconstriction and proliferation, while β2-AR stimulation promotes VSMC relaxation and inhibits migration—a dichotomy that highlights the receptor-dependent pleiotropy of sympathetic signaling. On lymphocytes, β2-AR signaling suppresses Th1 responses while promoting Th2 and Th17 polarization, depending on the local cytokine milieu. On endothelial cells, NE acts through α2-AR to modulate NO release and endothelial permeability. This cell-type- and receptor-specific signaling architecture underpins the context-dependent effects of sympathetic activation in cardiovascular disease (14). This direct signaling modulates immune cell functions, including the secretion of pro-inflammatory cytokines, and induces vasoconstriction, thereby influencing blood flow and pressure (15). Chronic elevation in sympathetic tone is a well-established core pathway linking psychological stress to increased cardiovascular risk, as it perpetuates a state of inflammation and vascular dysregulation (16). In contrast, the parasympathetic nervous system, particularly through the vagus nerve, provides a critical counterbalance via the cholinergic anti-inflammatory pathway. Vagus nerve stimulation leads to the release of acetylcholine (ACh), which binds to the α7 nicotinic acetylcholine receptor (α7nAChR) on macrophages, suppressing NF-κB activation and downstream pro-inflammatory cytokine production through the JAK2/STAT3 pathway. Beyond macrophages, α7nAChR is also expressed on T lymphocytes (where it modulates T-cell proliferation and cytokine secretion), dendritic cells (where it affects antigen presentation capacity), and endothelial cells (where it promotes NO production and maintains vascular barrier function). On VSMCs, ACh acts through muscarinic M3 receptors (M3R) to induce vasodilation via endothelium-dependent mechanisms, while M2 receptors on cardiac myocytes mediate parasympathetic control of heart rate. This receptor-specific cholinergic signaling establishes a multi-cellular anti-inflammatory and cardioprotective network that extends well beyond the canonical macrophage-centric model (14, 17). This interaction inhibits key pro-inflammatory signaling pathways, such as NF-κB activation, thereby suppressing the release of cytokines like TNF-α and IL-1β and exerting both local and systemic anti-inflammatory effects (18). This pathway is crucial for modulating the immune response in conditions such as sepsis and cardiovascular disease (19). Sensory neurons, which express channels like the transient receptor potential vanilloid 1 (TRPV1), serve a bidirectional role. They are responsible for afferent signaling, transmitting nociceptive and inflammatory signals from the periphery (e.g., from the heart or vasculature) to the central nervous system via neuropeptides such as substance P (SP) and calcitonin gene-related peptide (CGRP) (20). Conversely, these same sensory nerve terminals can release neuropeptides in the periphery, directly influencing vascular permeability, immune cell recruitment, and contributing to neurogenic inflammation (21, 22). This efferent function of sensory nerves establishes a local neuroimmune interface, where neuropeptides like CGRP can regulate adaptive immune responses, as seen in skin immunity (23). Thus, the integrated output from autonomic and sensory nerves forms a dynamic network that continuously adjusts immune activity and vascular tone, with imbalances in this neural regulation being central to the pathogenesis of inflammatory cardiovascular diseases, hypertension, and heart failure (24, 25).

Beyond the autonomic nervous system, the hypothalamic-pituitary-adrenal (HPA) axis represents another critical neuroendocrine output pathway that modulates immune function and cardiovascular homeostasis. Activation of the HPA axis in response to stress leads to the release of corticotropin-releasing hormone (CRH) from the hypothalamus, which stimulates pituitary adrenocorticotropic hormone (ACTH) secretion, ultimately driving adrenal glucocorticoid (primarily cortisol in humans) production. Glucocorticoids exert broad immunomodulatory effects by suppressing pro-inflammatory cytokine production, inhibiting NF-κB signaling, and promoting anti-inflammatory responses in innate and adaptive immune cells. In the cardiovascular system, chronic HPA axis dysregulation—characterized by sustained cortisol elevation—contributes to endothelial dysfunction, hypertension, and atherosclerosis progression. Importantly, glucocorticoids modulate the synthesis and release of catecholamines, establishing a bidirectional interaction between the HPA axis and sympathetic nervous system that amplifies neuroimmune signaling in CVD. Thus, the HPA axis functions as an indispensable humoral arm of the neuroimmune cardiovascular axis, translating psychosocial stress into sustained inflammatory and vascular pathology (26, 27).

### 2.2 Immune system response and signal feedback

The immune system is not merely a passive target of neural signals but an active participant in a bidirectional dialogue with the nervous system, capable of both receiving and generating feedback. A fundamental basis for this crosstalk is the widespread expression of receptors for neurotransmitters and neuropeptides on immune cells. Myeloid cells, including monocytes, macrophages, and dendritic cells, as well as lymphocytes such as T cells and B cells, express a variety of receptors including adrenergic receptors, cholinergic receptors, and receptors for neuropeptides like neuropeptide Y (25, 28). This allows them to directly “sense” and respond to neural output, adjusting their functional state—for instance, shifting from a pro-inflammatory to an anti-inflammatory phenotype—based on the local neurochemical milieu (14). Furthermore, activated immune cells themselves become potent sources of signaling molecules. They produce a plethora of cytokines (e.g., TNF-α, IL-1β, IL-6), chemokines, and reactive oxygen species (10). The actions of these cytokines are determined by specific receptor engagement on target cells: TNF-α signals through TNFR1 (ubiquitously expressed, mediating apoptosis and inflammation) and TNFR2 (predominantly on immune cells, mediating survival and proliferation); IL-1β engages the IL-1 receptor type 1 (IL-1R1) on endothelial cells, VSMCs, and cardiac myocytes to activate NF-κB and MAPK pathways; IL-6 signals through either classical cis-signaling (membrane-bound IL-6R, predominantly on hepatocytes and immune cells) or trans-signaling (soluble IL-6R, affecting all cell types, including endothelial cells and VSMCs)—the latter being particularly pathogenic in cardiovascular disease. These mediators have dual effects: they directly affect the function of neighboring vascular cells, such as endothelial cells and smooth muscle cells, and they can act on peripheral nerve endings or, via circulation, influence the central nervous system (29). This feedback can alter neural output, creating regulatory loops. For example, pro-inflammatory cytokines can sensitize sensory neurons, lowering their activation threshold and contributing to pain and dysautonomia, while also influencing central autonomic centers to increase sympathetic drive (30). The spleen emerges as a critical peripheral organ where this neuroimmune integration is particularly dense and functionally significant. It receives rich sympathetic noradrenergic innervation and houses a large reservoir of immune cells (31). Sympathetic nerves within the spleen can directly modulate the distribution, trafficking, and function of lymphocyte subsets, thereby exerting a systemic influence on inflammatory processes central to diseases like atherosclerosis and hypertension (32, 33). Studies show that splenic sympathetic activation can promote a pro-inflammatory immune profile, contributing to cardiovascular disease progression (31). Conversely, the spleen also contains specialized glial cells that ensheath sympathetic axons and are transcriptionally poised for immune communication, further highlighting its role as a neuroimmune hub (33). This intricate feedback system ensures that immune responses are finely tuned and context-dependent, but its dysregulation—characterized by chronic immune activation and impaired neural feedback—is a hallmark of numerous cardiovascular pathologies, from myocardial infarction to heart failure, where it exacerbates tissue damage and remodeling (25, 34).

In addition to innate immune cells, the adaptive immune system—particularly CD4+ T lymphocytes—plays an indispensable role in cardiovascular neuroimmunity (10, 11). T helper 1 (Th1) cells produce interferon-gamma (IFN-γ) and promote macrophage activation, while Th17 cells secrete interleukin-17 (IL-17) and drive neutrophilic inflammation; both subsets have been implicated in hypertension and atherosclerosis progression (35). Conversely, regulatory T cells (Tregs) exert immunosuppressive and atheroprotective functions by limiting excessive inflammation and maintaining immune tolerance (36). The sympathetic nervous system modulates this adaptive immune landscape through β2-adrenergic receptor signaling on T cells, which can suppress Th1 responses while promoting Th2 and Th17 polarization under certain contexts (14, 25). B cells also contribute to cardiovascular pathology through antibody production, antigen presentation, and cytokine secretion, with their function modulated by autonomic inputs (37). Thus, the adaptive immune system serves as both a sensor and effector of neural signals, translating sympathetic and parasympathetic tone into antigen-specific inflammatory or regulatory responses within the cardiovascular system.

### 2.3 Vascular system: the integrative and effector platform

**Figure 1:**
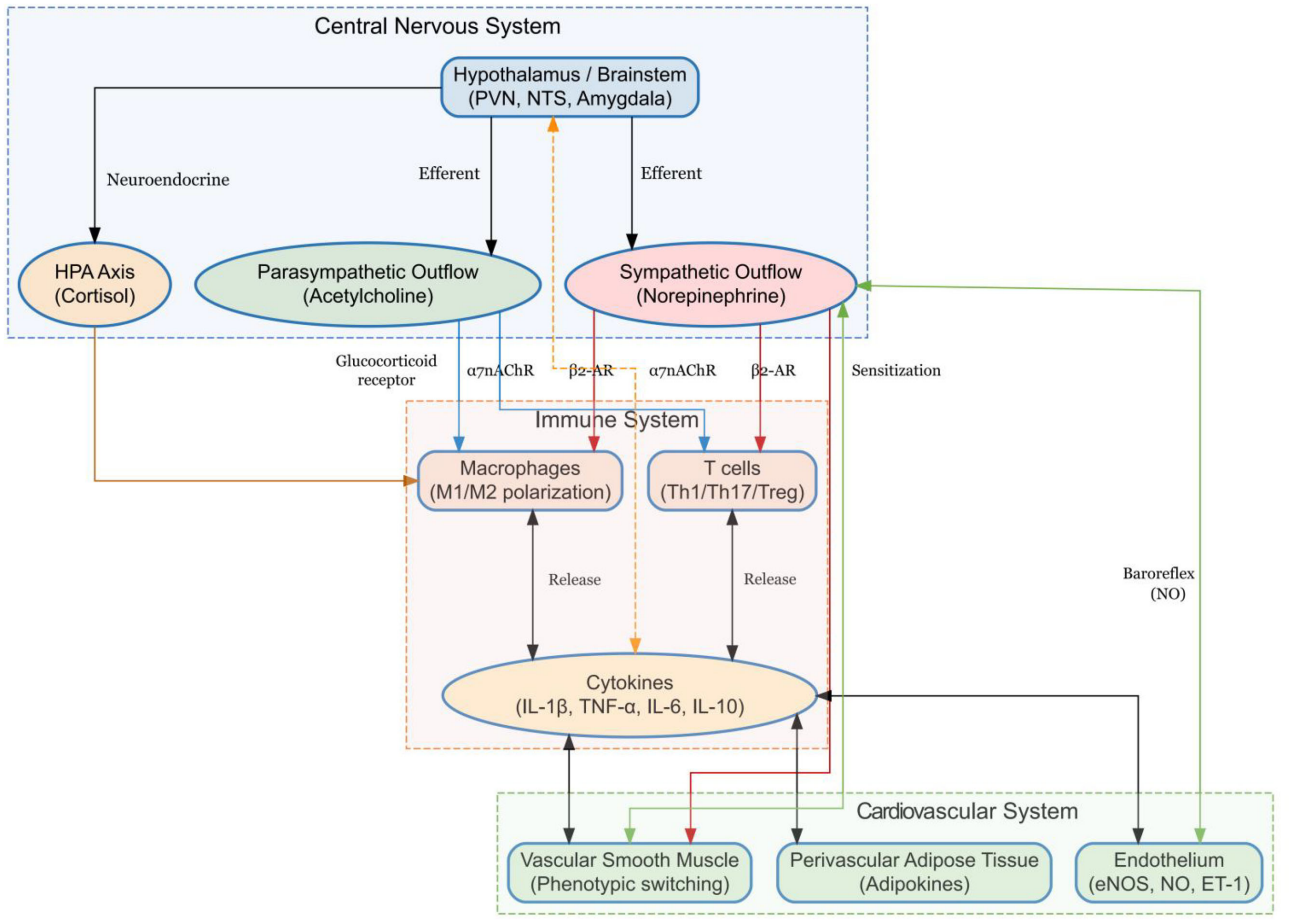
Schematic overview of the neural-immune-vascular axis. The image depicts the three core components and their bidirectional communication. The central nervous system (hypothalamus/brainstem) integrates signals and transmits them via sympathetic (NE), parasympathetic (ACh), and HPA axis (cortisol) outputs. These signals act on immune cells (macrophages, T cells) via β2-AR, α7nAChR, and glucocorticoid receptors, modulating cytokine release (IL-1β, TNF-α, IL-6, IL-10). Cytokines and neural signals converge on the vascular system, regulating endothelial function (eNOS, NO), VSMC phenotype, and PVAT inflammation. Bidirectional arrows indicate reciprocal feedback. α7nAChR, α7 nicotinic acetylcholine receptor; β2-AR, β2-adrenergic receptor; eNOS, endothelial nitric oxide synthase; HPA, hypothalamic-pituitary-adrenal; IL, interleukin; NE, norepinephrine; NO, nitric oxide; PVAT, perivascular adipose tissue; TNF-α, tumor necrosis factor-alpha; VSMC, vascular smooth muscle cell.

The vascular system serves as the primary effector and integrative platform where neural and immune signals converge to regulate hemodynamics, permeability, and inflammatory responses. At the center of this integration is the vascular endothelium. Endothelial cells express receptors for both neurotransmitters (e.g., adrenergic, cholinergic) and cytokines, allowing them to act as signal transducers (38). In addition to neurotransmitter receptors, endothelial cells express cytokine receptors (TNFR1, IL-1R1, IL-6R/gp130) and pattern recognition receptors (TLR2, TLR4), enabling them to integrate neural, inflammatory, and pathogen-derived signals. Downstream of these receptors, endothelial cells activate distinct signaling cascades—including NF-κB for inflammatory gene transcription, eNOS for NO production, and MAPK for proliferation and survival—each with specific functional outcomes. For example, β2-AR stimulation on endothelial cells promotes eNOS activation and vasodilation, whereas TNF-α signaling through TNFR1 induces adhesion molecule expression (ICAM-1, VCAM-1, E-selectin) and leukocyte recruitment. In response to cholinergic stimulation, endothelial cells release NO and hyperpolarizing factors, contributing to vasodilation and anti-inflammatory effects. This multi-receptor integration positions the endothelium as a critical signal transducer and effector of the neuro-immune-cardiovascular axis, translating both neural and immune inputs into vascular responses. In response to these inputs, endothelial cells synthesize and release key vasoactive substances such as nitric oxide (NO), prostaglandins, and endothelin. Endothelial nitric oxide synthase (eNOS)-derived NO is particularly crucial, as it mediates vasodilation, inhibits platelet aggregation and leukocyte adhesion, and modulates autonomic reflexes like the baroreflex, thereby playing a central role in maintaining vascular homeostasis and blood pressure control (38). The dysregulation of eNOS and a shift towards endothelial dysfunction are pivotal events in the pathogenesis of hypertension, atherosclerosis, and other cardiovascular diseases (38). Beyond the endothelium, a specialized anatomical and functional interface known as the perivascular neuro-immune synapse exists, particularly in the vascular adventitia and surrounding adipose tissue. Here, nerve terminals, including sympathetic and sensory fibers, are found in close apposition to immune cells such as macrophages (12, 25). This arrangement allows for rapid, localized, and precise signal exchange, similar to a synaptic connection. For instance, sympathetic neurotransmitters can directly activate adrenergic receptors on perivascular macrophages, influencing their cytokine production and phagocytic activity, which in turn affects vascular reactivity and the progression of conditions like atherosclerosis (12, 25). Conversely, immune cells in this niche can release mediators that affect adjacent nerve terminals. This perivascular neuroimmune communication is vital for coordinating micro-environmental inflammation and vascular tone. Its disruption is implicated in vascular pathologies; for example, in inflammatory bowel disease, impaired function of perivascular sensory nerves and altered signaling of their neurotransmitters (CGRP and substance P) contribute to mesenteric vascular dysfunction (21). Furthermore, in atherosclerosis, the expansion of axon networks in the diseased adventitia forms neuroimmune cardiovascular interfaces (NICIs), establishing a structural artery-brain circuit that controls disease progression (12). Thus, the vascular wall is not a passive conduit but a dynamic, innervated tissue where integrated neural and immune inputs determine vascular health, with endothelial cells and perivascular neuro-immune units acting as critical processors and amplifiers of these signals. The core components and bidirectional communication mechanisms of the neuro-immune-vascular axis are summarized schematically in **Figure 1**.

Beyond the endothelium, vascular smooth muscle cells (VSMCs) serve as critical transducers of neural and immune signals within the vascular wall. VSMCs exhibit remarkable phenotypic plasticity, switching from a contractile (quiescent) phenotype to a synthetic (activated) phenotype in response to inflammatory stimuli, neurotransmitters, and cytokines (39). This phenotypic switching is driven by pro-inflammatory cytokines (e.g., IL-1β, TNF-α) and growth factors (e.g., PDGF) released by infiltrating immune cells and activated endothelial cells (40). Sympathetic neurotransmitters such as norepinephrine can directly act on VSMCs via α1- and β2-adrenergic receptors, promoting proliferation, migration, and matrix remodeling—processes central to vascular stiffness and atherosclerosis.Conversely, activated VSMCs secrete chemokines and adhesion molecules that recruit immune cells into the vessel wall, establishing a feed-forward loop of vascular inflammation (41). This bidirectional neuroimmune-VSMC crosstalk underscores the VSMC as an active participant in the neuro-immune-cardiovascular axis, rather than a passive structural component (12, 25). Their key cellular players, primary mediators, and major pathological dysregulations are summarized in **Table 1**.

**Table 1:** Core components of the neural-immune-vascular axis in cardiovascular diseases.

Component	Key cells/structures	Main mediators	Pathological dysregulation
Neural output	Sympathetic/vagal/sensory nerves	NE, ACh, CGRP, SP	Sympathetic overdrive, vagal withdrawal
Immune system	Macrophages, T cells, microglia	IL-1β, TNF-α, IL-6, IL-10	Chronic inflammation, monocyte recruitment
Vascular system	Endothelium, pericytes, PVAT	NO, ET-1, ROS, adhesion molecules	Endothelial dysfunction, arterial stiffness

Across atherosclerosis, hypertension, and heart failure, three recurring mechanistic principles emerge from the neuro-immune-vascular framework. First, chronic sympathetic overdrive is a common driver—in each condition, sustained norepinephrine release promotes immune cell mobilization, pro-inflammatory cytokine production, and end-organ dysfunction, albeit through distinct downstream effectors (e.g., plaque macrophages in atherosclerosis, renal T cells in hypertension, cardiac β1-AR desensitization in heart failure) (3). Second, failure of the cholinergic anti-inflammatory pathway represents a shared vulnerability—reduced vagal tone and diminished α7nAChR signaling compromise the endogenous brake on inflammation across all three diseases (18). Third, a feed-forward inflammatory-sympathetic loop perpetuates disease progression—tissue injury generates cytokines that act on central autonomic centers, amplifying sympathetic outflow, which in turn fuels further inflammation (14). This vicious cycle operates at different anatomical sites but follows a conserved logic: the brain senses peripheral inflammation and responds by increasing sympathetic drive, creating a self-reinforcing circuit that drives disease chronicity. Recognizing these shared principles not only provides a unified framework for understanding disease pathogenesis but also identifies common therapeutic nodes that may be effective across multiple cardiovascular conditions.

## 3 Mechanisms of the neural-immune-cardiovascular axis in atherosclerosis

### 3.1 Sympathetic overdrive and plaque inflammation

Sympathetic overdrive, characterized by excessive norepinephrine (NE) release, is a critical driver of atherosclerotic plaque inflammation and progression. This heightened sympathetic activity, often associated with conditions like obstructive sleep apnea (OSA) and mental stress, directly promotes the recruitment and activation of immune cells within the arterial wall (42, 43). NE acts through β2-adrenergic receptors (β2-AR) on immune cells to facilitate the mobilization of myeloid cells, such as monocytes and neutrophils, from hematopoietic organs like the spleen and bone marrow into the circulation, thereby increasing their availability for infiltration into nascent plaques (44, 45). Once within the plaque microenvironment, NE signaling, potentially via α1-adrenergic receptors, can enhance the activation of the NLRP3 inflammasome in macrophages, leading to increased secretion of pro-inflammatory cytokines like interleukin-1β (IL-1β), a key mediator in the inflammatory cascade of atherosclerosis (46, 47). Single-cell transcriptomic studies have identified a specific subset of sympathetic-responsive inflammatory macrophages within atherosclerotic plaques that are distinct from classical lipid-laden foam cells. These sympathetic-responsive macrophages co-express adrenergic receptors and exhibit enhanced inflammatory gene signatures. In contrast, other plaque-resident macrophage subsets, such as those expressing Trem2 and Gpnmb (associated with lipid-associated macrophages), appear less responsive to NE stimulation. Thus, the pro-inflammatory effects of sympathetic signaling on macrophages are not uniform but target specific transcriptional and functional subsets within the plaque microenvironment. This neuroimmune interaction establishes a vicious cycle where inflammation begets further sympathetic activation, as seen in the context of metabolic dysfunction-associated steatotic liver disease (MASLD), which is linked to atherosclerosis through mechanisms including sympathetic nervous system activation (48). The consequences of this sustained inflammatory state are profound for plaque stability. Sympathetic activity promotes the expression of matrix metalloproteinases (MMPs) by vascular cells and infiltrating macrophages, which degrade the extracellular matrix of the fibrous cap, the structure that maintains plaque integrity (46). Concurrently, NE can inhibit collagen synthesis, further weakening the cap’s structural support. This dual action of enhancing degradation and suppressing repair significantly increases the risk of plaque rupture, the precipitating event for acute coronary syndromes like myocardial infarction (49). Furthermore, the sympathetic nervous system intricately modulates lymphocyte function within plaques, skewing the immune response towards a pro-atherogenic phenotype. NE influences the balance between pro-inflammatory T helper 1 (Th1) cells and anti-inflammatory regulatory T cells (Tregs), favoring Th1 responses that promote macrophage activation and cytokine production while suppressing the protective, immunosuppressive functions of Tregs (45). This imbalance is exacerbated by conditions like chronic social isolation, which disrupts hypothalamic signaling and can aggravate atherosclerosis, though interestingly, this particular pathway may operate independently of changes in sympathetic nervous activity (50). The spleen emerges as a pivotal neuroimmune interface in this process, where sympathetic efferents can modulate the egress of various immune cells, including T cells and monocytes, into the circulation, directly feeding the inflammatory milieu of atherosclerotic lesions (51, 52). Therapeutic strategies that target this sympathetic overdrive, such as renal denervation (RDN), have demonstrated efficacy in experimental models by reducing splenic sympathetic activity, curtailing the production and release of monocytes and neutrophils, and ultimately mitigating plaque inflammation and size (44). This underscores the sympathetic nervous system not merely as a bystander but as a central regulator within the neuro-immune axis that governs the inflammatory pathogenesis and destabilization of atherosclerotic plaques.

An emerging layer of complexity in neuroimmune regulation of atherosclerosis involves clonal hematopoiesis of indeterminate potential (CHIP)—an age-related phenomenon in which somatic mutations in hematopoietic stem cells (most commonly in TET2, DNMT3A, ASXL1, and JAK2) drive the clonal expansion of mutant leukocytes (53). CHIP mutations confer a pro-inflammatory phenotype to monocytes and macrophages, enhancing NLRP3 inflammasome activation and IL-1β production, and have been established as an independent risk factor for atherosclerotic cardiovascular disease. Importantly, emerging evidence suggests that CHIP intersects with the neuroimmune axis: sympathetic nervous system activation may promote the expansion of CHIP clones, while CHIP-derived inflammatory monocytes amplify the vascular inflammatory response to sympathetic overdrive (51). This neuroimmune-CHIP crosstalk represents a previously unrecognized mechanism linking age-related hematopoietic changes, autonomic dysfunction, and atherosclerosis progression. Therapeutic strategies targeting CHIP—such as anti-inflammatory agents or IL-1β inhibitors—may offer novel opportunities to break this neuroimmune-hematopoietic cycle [speculative/preclinical; no human data available to date] (54).

### 3.2 Impaired function of the cholinergic anti-inflammatory pathway

**Figure 2:**
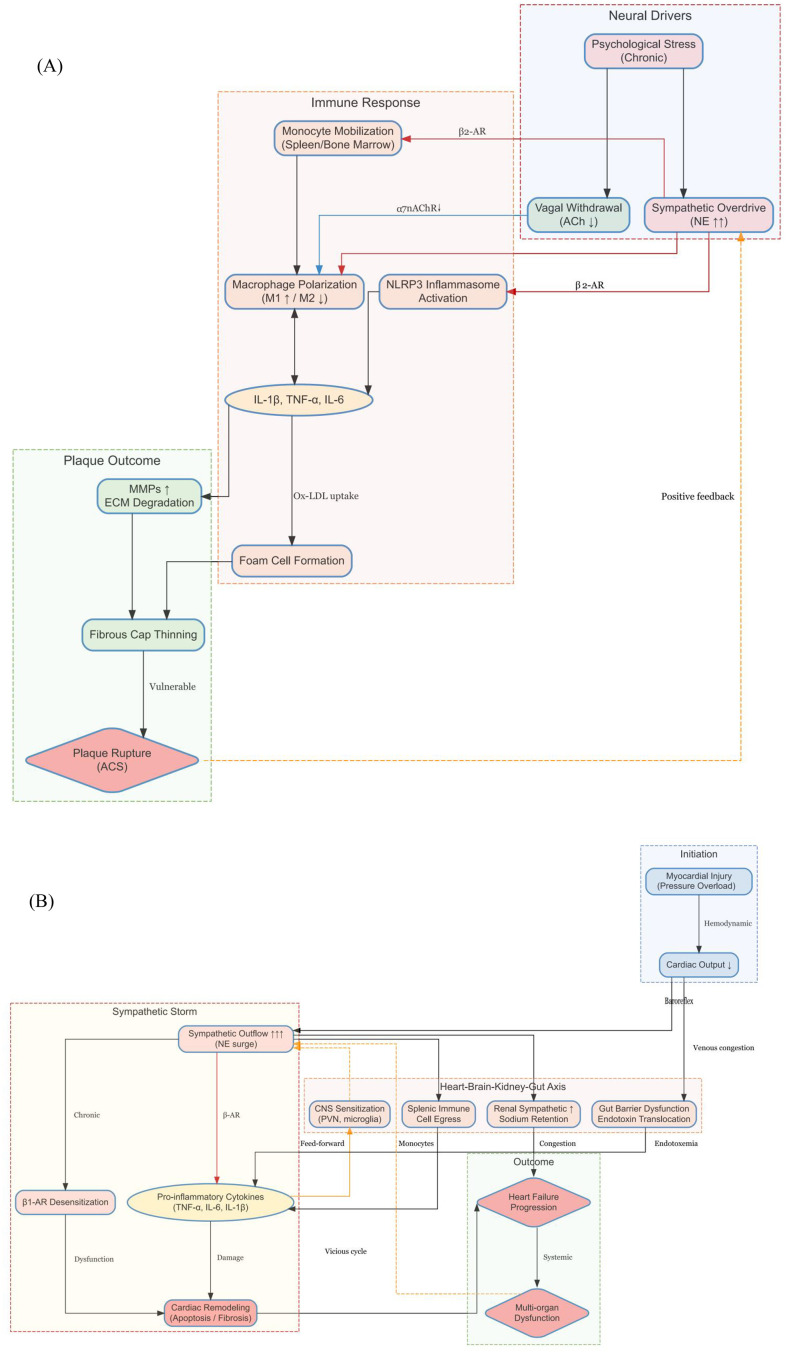
Disease-specific mechanistic maps of the neural-immune-vascular axis. **(A)** In atherosclerosis, chronic stress drives sympathetic overdrive (NE ↑↑) and vagal withdrawal (ACh ↓), promoting monocyte mobilization, NLRP3 inflammasome activation, and the adoption of pro-inflammatory macrophage states. Pro-inflammatory cytokines (IL-1β, TNF-α, IL-6) enhance foam cell formation, MMP upregulation, and ECM degradation, leading to fibrous cap thinning and plaque rupture. Dashed arrow indicates positive feedback to sympathetic activation. **(B)** In heart failure, reduced cardiac output triggers sympathetic storm, causing β1-AR desensitization, cytokine release, and cardiac remodeling (apoptosis, fibrosis). The heart-brain-kidney-gut axis amplifies sympathetic outflow through CNS sensitization, renal sodium retention, and gut endotoxin translocation. Splenic immune egress fuels systemic inflammation. Dashed arrows indicate feed-forward vicious cycles. ACS, acute coronary syndrome; AR, adrenergic receptor; ECM, extracellular matrix; MMP, matrix metalloproteinase; NLRP3, NOD-like receptor family pyrin domain containing 3; PVN, paraventricular nucleus.

In contrast to the pro-inflammatory effects of sympathetic overdrive, the parasympathetic nervous system, primarily via the vagus nerve, exerts potent anti-inflammatory control through the cholinergic anti-inflammatory pathway. However, in the context of aging and cardiovascular diseases like atherosclerosis, this protective reflex is often impaired, characterized by reduced vagal tone and diminished parasympathetic activity (17). This decline in vagal output compromises a critical homeostatic mechanism that normally curbs excessive inflammation, thereby contributing to a persistent low-grade inflammatory state that fuels endothelial dysfunction and atherogenesis (17). The efferent arm of this reflex acts through the α7-nicotinic acetylcholine receptor (α7nAChR), which is expressed on macrophages, monocytes, dendritic cells, T cells, and endothelial cells (17). Activation of α7nAChR on splenic macrophages, a process that involves a complex circuit where vagal efferents activate noradrenergic splenic nerves, which then stimulate acetylcholine release from splenic T cells, ultimately inhibits the nuclear translocation of NF-κB and attenuates the release of pro-inflammatory cytokines such as tumor necrosis factor-alpha (TNF-α) (51). This vagosplenic axis is crucial for modulating systemic inflammation, and its dysfunction means the body loses a key brake on inflammatory responses within the vasculature and atherosclerotic plaques. The therapeutic potential of enhancing this pathway is substantial and has been validated in experimental studies. Pharmacological agonists of the α7nAChR or direct electrical stimulation of the vagus nerve (VNS) have been shown to significantly reduce atherosclerotic plaque burden and inflammation in animal models, confirming that augmenting cholinergic signaling holds promise as an anti-inflammatory strategy for cardiovascular disease (55). This approach targets the neuroimmune cardiovascular interfaces (NICIs), specialized sites of communication between nerves and immune cells in the vascular adventitia, which are restructured in advanced atherosclerosis (46). Furthermore, non-invasive techniques like device-guided slow breathing, which can enhance parasympathetic activity and baroreflex sensitivity, have demonstrated clinical benefits by reducing both blood pressure and systemic inflammatory markers like TNF-α in hypertensive patients, highlighting a practical intervention that may partially restore cholinergic anti-inflammatory tone (56). The impairment of this pathway is also implicated in the pathophysiology of heart failure, where chronic sympathetic upregulation is a hallmark, and neuromodulation therapies like acupuncture are being explored for their potential to rebalance autonomic function (57). The spleen’s role is again central, as it is a major target for this neuromodulation; interventions like auricular tragus vagal stimulation and remote ischemic conditioning are thought to exert acute cardioprotective effects, at least in part, by activating the vagosplenic axis (51). Conversely, splenectomy, which disrupts this neuroimmune organ, is associated with increased long-term mortality from ischemic heart disease in humans, underscoring the physiological importance of the spleen in mediating the cholinergic anti-inflammatory response within the cardiovascular system (51). Therefore, the dysfunction of the cholinergic anti-inflammatory pathway represents a significant vulnerability in cardiovascular health, and strategies aimed at its restoration—whether through pharmacological α7nAChR activation, vagus nerve stimulation, or lifestyle interventions—constitute a promising frontier for developing novel anti-inflammatory therapies that move beyond traditional lipid-lowering approaches. The key neuroimmune mechanisms driving atherosclerotic plaque inflammation and vulnerability are illustrated in **Figure 2A**.

## 4 The role of the neural-immune-vascular axis in the pathogenesis and development of hypertension

### 4.1 Central sympathetic activation and immune priming

Chronic stress or high-salt diets initiate a cascade of neuroimmune events that are central to the development of cardiovascular pathologies. These stimuli activate specific central nuclei, such as the hypothalamic paraventricular nucleus (PVN), leading to increased sympathetic outflow (58). This central sympathetic activation is a critical driver, as it not only increases peripheral sympathetic nerve activity but also activates systemic and local tissue renin-angiotensin systems (RAS) (10). The autonomic nervous system (ANS), hypothalamic-pituitary-adrenal (HPA) axis, and immune system are anatomically and functionally interconnected, coordinating the body’s response to perceived and systemic stress signals (59). Within this network, sympathetic activity exerts context-dependent effects on immune signaling, which can be either pro- or anti-inflammatory depending on factors like receptor subtype and timing (59). A key mediator in this process is angiotensin II (Ang II), a potent pro-inflammatory agent released upon RAS activation. Ang II promotes the infiltration of immune cells into critical target organs like blood vessels and the kidneys, establishing a direct link between neurohormonal activation and peripheral inflammation (10). The renin-angiotensin-aldosterone system (RAAS) functions as a critical neuroimmune integrator that bridges this sympathetic activation to downstream immune and vascular pathology. Angiotensin II (Ang II), the primary effector of RAAS, exerts potent immunomodulatory effects that extend well beyond its classical vasoconstrictive actions. Ang II promotes T cell activation and differentiation toward pro-inflammatory Th1 and Th17 phenotypes, while suppressing regulatory T cell (Treg) function (60). It also induces dendritic cell maturation and enhances their antigen-presenting capacity (61), amplifying adaptive immune responses within the vasculature and kidney. In macrophages, Ang II drives polarization toward a pro-inflammatory functional state characterized by enhanced production of IL-1β, TNF-α, and reactive oxygen species (62). This Ang II-driven immune activation is both a cause and consequence of hypertension: sympathetic activation stimulates renal renin release, increasing circulating Ang II, which in turn activates immune cells that promote vascular inflammation, sodium retention, and further sympathetic excitation. Thus, RAAS operates at the nexus of neurohumoral and immune signaling, making it a canonical example of neuroimmune integration in hypertension and a prime therapeutic target. Furthermore, emerging research reveals a sophisticated “neuroimmune dialogue” within the brain itself. Salt loading or Ang II can activate the NLRP3 inflammasome in brain regions such as the hypothalamus, leading to the production of interleukin-1β (IL-1β) (10). This central inflammatory signal further exacerbates sympathetic excitation and contributes to hypertension, creating a vicious cycle where peripheral signals feed back to amplify central dysregulation (10). The role of central immune cells, particularly microglia, in this dialogue is paramount. For instance, after myocardial infarction, microglia in the PVN become activated and mediate a neuroimmune response that regulates sympathetic activity and subsequent pathological cardiac remodeling (63). Depletion of these central microglia attenuates this neuroimmune response, decreases sympathetic drive, and improves cardiac outcomes, highlighting the PVN as a crucial hub for integrating immune and neural signals in cardiovascular disease (63). This central communication extends beyond the brain, as the peripheral nervous system (PNS) engages in bidirectional crosstalk with lymphoid tissues and organs, forming neuroimmune cell units that regulate local immune responses (28). The sympathetic neurotransmitter norepinephrine (NE) plays a complex role in this interplay. The sympathetic system also mediates an anti-inflammatory reflex; for example, endotoxin challenge can activate an adrenomedullary pathway involving adrenaline (epinephrine) to promote hyperglycemia and inhibit tumor necrosis factor (TNF) secretion, showcasing a systemic neuroendocrine-immune defense mechanism (64). This intricate web of communication underscores the concept of the “brain-organ axis,” where psychological stress, via HPA and sympathetic activation, modulates peripheral immunity in an organ-specific manner, promoting pro-inflammatory mediator release and altering immune surveillance (65). Thus, the activation of central sympathetic pathways, through both direct neural efferents and systemic hormonal cascades, serves as a fundamental mechanism for immune system priming, setting the stage for the peripheral vascular and renal dysfunction characteristic of cardiovascular diseases.

### 4.2 Peripheral immune-mediated vascular dysfunction and renal injury

The efferent signals from the activated central sympathetic and neuroimmune axis manifest in the periphery as immune-mediated damage to the vasculature and kidneys, which are key target organs in hypertension and related cardiovascular diseases. Activated T cells, particularly those producing interferon-gamma (IFN-γ) and interleukin-17 (IL-17), infiltrate the perivascular adipose tissue (PVAT) and the kidneys (10). These infiltrating lymphocytes are potent drivers of vascular oxidative stress. They promote vascular smooth muscle cells to produce excess superoxide, which scavenges nitric oxide (NO), leading to a marked decrease in NO bioavailability (10). The resultant impairment in endothelial-dependent vasodilation and enhanced vasoconstriction is a hallmark of vascular dysfunction. This process is exacerbated in conditions like atherosclerosis, where sympathetic overactivation, measured by plasma norepinephrine levels, correlates positively with the accumulation of circulatory myeloid cells and inflammatory cytokines, establishing a direct link between the sympathetic nervous system (SNS) and vascular inflammation (44). The kidney is another critical battlefield. Renal sympathetic nerve activity (RSNA) is increased in hypertension and plays a critical role in its perpetuation and the development of hypertensive kidney disease (66). Norepinephrine released from renal sympathetic nerves modulates not only renin release, vascular resistance, and sodium handling but also the local immune cell response (66). Within the renal interstitium, macrophages play a pivotal role in sodium homeostasis and blood pressure regulation. These renal macrophages can produce cytokines such as IL-1β and tumor necrosis factor-alpha (TNF-α), which influence the expression and activity of the epithelial sodium channel (ENaC) in renal tubules (10). By promoting sodium reabsorption, these immune cells directly contribute to salt retention and the pathogenesis of salt-sensitive hypertension. The broader neuroimmune control of inflammation is also evident in acute kidney injury (AKI), where the inflammatory reflex pathway, including the cholinergic anti-inflammatory pathway (CAP), and renal sympathetic nerve activity modulate kidney function and inflammation (67). The spleen emerges as a crucial immune organ in this peripheral response. In atherosclerosis, renal denervation (RDN) reduces sympathetic activity and attenuates disease progression not only by local renal effects but also by suppressing the production of neutrophils and monocytes in the spleen, indicating a neuroimmune circuit that regulates hematopoietic output (44). This aligns with the concept of a “cardiopulmonary neuro-immune reflex,” where afferent stress signals from the heart and lungs to the brain lead to efferent sympathetic outflow that modulates immune cell dynamics in the bone marrow and spleen, promoting myeloid skewing and inflammatory macrophage expansion (68). In ischemic heart disease, the spleen releases various immune cells in temporally distinct patterns to execute innate and adaptive immune processes in atherosclerotic plaques and ischemic myocardium (51). Furthermore, neuromodulation through the vagosplenic axis, involving noradrenergic splenic nerve activation, can attenuate cytokine secretion from splenic macrophages and has been shown to protect against ischemia-reperfusion injury (51). The perivascular adipose tissue (PVAT) itself becomes a significant source of pro-inflammatory adipokines in cardiometabolic diseases, attracting immune cell infiltration and exacerbating vascular smooth muscle cell proliferation and endothelial dysfunction (69). This local vascular inflammation is a key component of endothelial dysfunction, an early manifestation of atherosclerosis that can be induced by mental stress through increased sympathetic nervous system activity, cortisol-mediated inhibition of NO synthesis, and elevated pro-inflammatory cytokines (43). The resulting systemic inflammation activates a neural-hematopoietic-arterial axis, involving bone marrow activation and leukocyte migration to the arterial wall (43). In the context of metabolic dysfunction, such as in obesity and diabetes, this peripheral immune dysregulation is further amplified. Hyperleptinemia, common in obesity, promotes the secretion of pro-inflammatory cytokines like IL-6, TNF-α, and IL-17, which not only drive chronic inflammatory disorders but also contribute to atherosclerosis and cardiovascular complications (70). The dysregulated sympathetic nervous system activity associated with these conditions is a pivotal factor in disease progression (57). Ultimately, the peripheral immune response, orchestrated by central neural commands and local neuroimmune interactions, directly causes vascular oxidative stress, endothelial dysfunction, and renal sodium handling abnormalities, forming the effector arm of the neuro-immune-cardiovascular axis in cardiovascular disease pathogenesis.

## 5 The neural-immune-cardiovascular axis and the vicious cycle of heart failure

### 5.1 Sympathetic storm and systemic inflammation in chronic heart failure

#### 5.1.1 Pathological transformation of compensatory sympathetic activation

In the initial stages of heart failure, sympathetic nervous system activation serves as a compensatory mechanism to maintain cardiac output. However, chronic and excessive activation leads to pathological consequences, including desensitization of myocardial β1-adrenergic receptors, cardiomyocyte apoptosis, fibrosis, and the stimulation of pro-inflammatory cytokine production both within the heart and systemically. This maladaptive neurohormonal response is a central feature of heart failure progression. The sympathetic overdrive is not merely a marker of disease severity but a direct contributor to adverse cardiac remodeling and systemic inflammation. For instance, in structural heart disease, conditions like electrical storm, characterized by recurrent ventricular arrhythmias, are associated with a significant increase in mortality and heart failure hospitalization, underscoring the lethal potential of unchecked sympathetic activity (71). The pathophysiology of stress-induced cardiomyopathies, such as Takotsubo syndrome, further illustrates this link. In this condition, acute stress and subsequent excessive sympathetic activation with high catecholamine levels trigger reactive oxygen species generation, inflammatory cytokine release, and endothelial injury, initiating the disease process (72). Similarly, in thyrotoxicosis, excess thyroid hormone alters adrenergic receptor function, increasing sympathetic tone and contributing to arrhythmias like atrial fibrillation, demonstrating the interplay between endocrine dysregulation, sympathetic activation, and cardiac dysfunction (73). This sustained catecholamine surge promotes a pro-inflammatory state, creating a vicious cycle where inflammation further stimulates sympathetic outflow. Evidence from chronic heart failure patients shows a direct correlation between the intensity of systemic inflammation, measured by markers like C-reactive protein (CRP) and interleukin-6 (IL-6), and the clinical severity of the condition (74). Systemic inflammation-based prognostic scores, such as the Glasgow Prognostic Score (GPS) and the Systemic Immune-Inflammation Index (SII), are strong independent predictors of long-term mortality in chronic heart failure, validating the clinical relevance of this interaction (75, 76). Pharmacological interventions that modulate this axis, such as sodium-glucose co-transporter 2 inhibitors (SGLT2i), have demonstrated anti-inflammatory effects in heart failure patients, including reductions in fibrinogen levels, irrespective of ejection fraction phenotype, highlighting a potential mechanism for their cardioprotective benefits (77). Systemic inflammation is prevalent across the entire spectrum of heart failure, affecting approximately half of all patients, and is strongly associated with comorbidities like obesity, diabetes, and chronic kidney disease, which further fuel the neuro-inflammatory axis (78). The mineralocorticoid receptor antagonist finerenone also targets inflammation and fibrosis, showing benefits in patients with chronic kidney disease and type 2 diabetes, conditions often comorbid with heart failure (79). Predictive models integrating systemic inflammatory markers with traditional risk factors have shown satisfactory performance in forecasting adverse outcomes in chronic heart failure, emphasizing the prognostic value of assessing inflammation (80). Ultimately, the transition from compensatory sympathetic activation to a pathological “sympathetic storm” is a critical driver of myocardial injury, systemic inflammation, and disease progression in chronic heart failure.

#### 5.1.2 “Heart-brain-kidney” axis and inflammation

In heart failure, reduced cardiac output and venous congestion activate complex reflex pathways involving the “heart-brain-kidney” axis, which further exacerbates sympathetic outflow and triggers a systemic inflammatory response. The decline in cardiac output activates baroreceptors and cardiopulmonary receptors, leading to increased sympathetic signaling from the central nervous system to the kidneys, resulting in renal sympathetic nerve activation, sodium retention, and worsened congestion. This sympathetic activation is closely intertwined with inflammation. Circulating pro-inflammatory cytokines can act on the brain to further stimulate sympathetic nervous system activity, creating a feed-forward loop (81). For example, dysregulation of hypoxia-inducible factor 1α in the sympathetic nervous system has been shown to accelerate adverse myocardial remodeling and cardiac dysfunction in diabetic cardiomyopathy, linking neural dysregulation to inflammatory structural changes (82). Concurrently, venous congestion, particularly in the splanchnic circulation, leads to intestinal wall edema and barrier dysfunction. This disruption of the gut barrier allows for bacterial endotoxin translocation from the intestinal lumen into the systemic circulation, a process known as endotoxemia, which potently triggers a systemic inflammatory response (74). This gut-derived inflammation exacerbates myocardial injury and contributes to worsening renal function, forming a cardiorenal syndrome. The lymphatic system, crucial for fluid homeostasis and immune cell trafficking, becomes dysfunctional under conditions of chronic venous congestion and inflammation, failing to adequately drain interstitial fluid and inflammatory mediators, thereby perpetuating tissue edema and inflammation (83). This creates a vicious cycle where heart failure leads to gut and lymphatic dysfunction, which in turn amplifies systemic inflammation, further damaging the heart and kidneys. The sympathetic nervous system and inflammation exhibit a bidirectional relationship within this axis. Increased sympathetic activity promotes inflammation through the release of catecholamines that act on adrenergic receptors on immune cells, while inflammatory cytokines can stimulate central sympathetic centers (14). Sodium-glucose cotransporter 2 inhibitors may exert part of their benefit by modifying the intrarenal environment (e.g., reducing tissue hypoxia, inflammation, oxidative stress) to suppress afferent renal nerve activity, thereby reducing central sympathetic outflow to the heart and kidneys (84). This neuro-immune interaction is also evident in the regulation of specific inflammatory pathways, such as the NLRP3 inflammasome, which is implicated in cardiovascular diseases and can be modulated by autonomic nervous system activity, including vagus nerve stimulation (85). The central role of sympathetic activation in heart failure, involving both central nervous system drive and impaired reflex control, is well-established and contributes to adverse outcomes in multiple organ systems, including the brain, heart, and kidneys (86). The intricate connection between diabetes and heart failure is another manifestation of this axis, where chronic sympathetic upregulation and inflammation are pivotal pathophysiological links (57). The gut microbiome contributes to this inflammatory milieu in heart failure; dysbiosis and increased intestinal permeability facilitate the translocation of microbes and metabolites that induce systemic inflammation, further deteriorating cardiac function (87). Neuromodulation therapies targeting the autonomic nervous system aim to restore sympathetic-parasympathetic balance and reduce systemic inflammation, representing a complementary approach to standard heart failure treatments (88). Importantly, some therapeutic agents, like the nonsteroidal mineralocorticoid receptor antagonist esaxerenone, can lower blood pressure and provide organ protection without inducing reflex sympathetic activation, which is a common drawback of other vasodilators (89). Antidiabetic drugs can also interact with this heart-brain axis, with some classes like SGLT2 inhibitors showing benefits for both cardiovascular and cognitive outcomes potentially through anti-inflammatory mechanisms, while others may have complex effects on sympathetic activity (90). The brain-heart axis is crucial in conditions like ischemic heart disease, where autonomic imbalance (increased sympathetic and decreased parasympathetic tone) promotes inflammation, vasoconstriction, and ischemia, making neural modulation therapies a potential future avenue (91). In heart failure with preserved ejection fraction (HFpEF), enhanced sympathetic vasomotor tone, potentially driven by inflammatory activation of the sympathetic nervous system, is implicated in the macro- and microvascular dysfunction characteristic of the disease (92). Furthermore, inflammation plays a key role in the pathophysiology of increased renal sodium avidity in heart failure, contributing to diuretic resistance and congestion (93). The concept of acute-on-chronic inflammation is critical, as patients with underlying hypertension and heart failure are at increased risk of organ failure, such as renal failure, during acute illnesses, with biomarkers like CRP signaling excessive inflammation (94). At a molecular level, neurogenic inflammatory cascades, such as those mediated by the mechanosensitive channel Piezo1 in thoracic dorsal root ganglia, can exacerbate ventricular remodeling after myocardial infarction by releasing inflammatory mediators like IL-6, highlighting a direct neuronal-immune signaling axis in cardiac pathology (95). Thus, the “heart-brain-kidney” axis, through its integration of hemodynamic signals, sympathetic reflexes, gut barrier integrity, and immune activation, is a fundamental pathway through which heart failure propagates systemic inflammation, driving multi-organ dysfunction and disease progression.

### 5.2 Immune cell involvement in myocardial remodeling

#### 5.2.1 Imbalance in macrophage phenotype switching

Following myocardial infarction or under conditions of chronic pressure overload, the polarization balance of cardiac macrophages shifts detrimentally, driving adverse myocardial remodeling. Both resident cardiac macrophages and those recruited from the circulation are involved. In a healthy state or during the reparative phase, macrophages exhibiting a reparative functional state—characterized by tissue repair, anti-inflammatory cytokine release (e.g., IL-10), and debris clearance—are predominant. However, in the failing heart, there is a persistent imbalance favoring pro-inflammatory macrophage states over reparative subsets. These pro-inflammatory macrophages continuously release potent cytokines such as IL-1β, TNF-α, and IL-6. This sustained inflammatory milieu directly promotes pathological processes: TNF-α and IL-1β can induce cardiomyocyte hypertrophy and apoptosis, while transforming growth factor-beta (TGF-β) and other mediators from activated macrophages and myofibroblasts drive excessive interstitial collagen deposition and fibrosis, impairing cardiac compliance and function. This chronic, low-grade inflammation is a hallmark of heart failure progression. The interaction between autonomic nerves and macrophages is a key mechanistic link in cardiovascular disease pathophysiology, where sympathetic signaling can influence macrophage recruitment and polarization (25). In heart failure with preserved ejection fraction (HFpEF), systemic chronic inflammation leads to an imbalance of immune cells, including macrophages, within the heart, resulting in progressive fibrosis and dysfunction (96). The role of inflammation is central, as it contributes to endothelial dysfunction, oxidative stress, and the remodeling processes in both heart failure with reduced and preserved ejection fraction (97). Natural compounds like sclareol have been shown to protect against angiotensin II-induced cardiac remodeling and inflammation in mice, in part by inhibiting MAPK signaling pathways that drive pro-inflammatory responses in cardiomyocytes and likely immune cells (98). Inflammation is a multifaceted process in heart failure, involving the activation of both innate and adaptive immune systems, with macrophages as central innate immune effectors (99). In autoimmune conditions like rheumatoid arthritis, the associated chronic systemic inflammation significantly increases the risk of left ventricular diastolic dysfunction, a process where macrophage-mediated inflammation likely plays a contributory role (100). The spleen and bone marrow are increasingly recognized as key organs in HFpEF pathophysiology, contributing to systemic inflammation and immune cell production, including monocytes that can differentiate into cardiac macrophages (35, 96). Neutrophil extracellular traps (NETs), another component of innate immunity, are implicated in the pathogenesis of heart failure through mechanisms involving oxidative stress and perpetuation of inflammation, which can influence the cardiac macrophage environment (101). The emerging field of immunometabolism highlights the bidirectional crosstalk between immune cells (like macrophages) and cardiac parenchymal cells, where metabolic shifts in immune cells influence their pro- or anti-inflammatory polarization, contributing to adverse remodeling (102). Dietary fats can influence this inflammatory resolution; for example, docosahexaenoic acid (DHA) promotes the biosynthesis of specialized pro-resolving mediators and increases the number of resolving macrophages in a splenocardiac manner post-myocardial infarction, improving outcomes in heart failure (103). Iron metabolism is also linked to inflammation and macrophage function; in pressure overload models, iron chelation reduced cardiac inflammation and hypertrophy, suggesting a role for iron in macrophage-mediated inflammatory pathways (104). The brain influences systemic inflammation through neuroimmune circuits that involve organs like the bone marrow and spleen, ultimately affecting immune cell recruitment, including macrophages, to the heart (5, 105). Sleep apnea, a condition associated with increased cardiac risk, induces sympathetic activation and systemic inflammation, which can contribute to the pro-inflammatory macrophage polarization seen in heart failure (106). The NLRP3 inflammasome, a key inflammatory platform, is activated in macrophages and other cells in cardiovascular disease and can be regulated by the autonomic nervous system (85). The gut-heart axis also contributes to this inflammatory environment; dysbiosis and increased intestinal permeability lead to endotoxemia and metabolite translocation, which can activate systemic and cardiac macrophages, fueling inflammation that deteriorates cardiac function (87). Therefore, the failure to resolve inflammation due to an imbalance in macrophage phenotype switching from reparative toward pro-inflammatory functional states is a fundamental mechanism driving cardiomyocyte dysfunction, death, and fibrotic scarring in the remodeling heart.

#### 5.2.2 Lymphocyte-mediated autoimmune response

Emerging evidence indicates that myocardial injury can expose intracellular cardiac antigens, such as myosin, triggering an adaptive immune response that contributes to chronic inflammation and progression of heart failure. This process involves the activation of antigen-specific T lymphocytes and the production of autoantibodies. Following an initial insult like myocardial infarction or myocarditis, cardiac cell necrosis releases self-antigens that are normally sequestered from the immune system. These antigens can be presented by antigen-presenting cells (e.g., dendritic cells, macrophages) to T cells, potentially breaking immunological tolerance. Activated CD4+ T helper cells can promote B cell differentiation into plasma cells that produce autoantibodies against cardiac proteins, while cytotoxic CD8+ T cells may directly attack cardiomyocytes. This autoimmune response leads to a state of persistent, low-grade myocardial inflammation even after the initial injury has subsided, promoting ongoing cardiomyocyte apoptosis, interstitial fibrosis, and adverse ventricular remodeling. The chronic inflammatory state inherent to heart failure itself can foster a microenvironment conducive to such autoimmune phenomena. The relationship between cancer and heart failure provides a parallel, as both conditions share common pathological origins involving chronic inflammation, and heart failure-induced sympathetic and renin-angiotensin-aldosterone system activation can promote processes like cancer development, hinting at systemic immune dysregulation (107). Inflammation is a key driver in the pathophysiology of heart failure, with both innate and adaptive immunity playing significant roles (99). The immunometabolism of heart failure involves complex interactions where adaptive immune cells, like T and B lymphocytes, are recruited and polarized, contributing to the inflammatory milieu that drives adverse remodeling (102). For instance, in chronic heart failure models, docosahexaenoic acid (DHA) supplementation was associated with a higher number of T regulatory cells (Tregs, marked by Foxp3+) in the myocardium of heart failure survivors, suggesting an attempt at immune regulation and highlighting the involvement of lymphocyte subsets in the cardiac inflammatory landscape (103). The brain can influence systemic inflammation through neuroimmune circuits, which may modulate adaptive immune responses affecting the heart (5). Furthermore, the interaction between the autonomic nervous system and inflammation is bidirectional; sympathetic activation can influence immune cell function, including lymphocytes, while inflammatory cytokines can stimulate sympathetic centers (14). Neuromodulation therapies aim to restore autonomic balance, which may indirectly modulate these maladaptive immune responses (88). The gut microbiota also plays a role in systemic inflammation in heart failure, and dysbiosis can influence autoimmune mechanisms through molecular mimicry or bystander activation, contributing to the inflammatory burden that worsens cardiac function (87). Thus, lymphocyte-mediated autoimmunity represents a specific and sustained inflammatory pathway that extends beyond the initial injury, perpetuating myocardial damage and fibrosis, and represents a potential therapeutic target for mitigating the progression of chronic heart failure. The neuroimmune circuits and multi-organ interactions that perpetuate the vicious cycle of heart failure progression are depicted in **Figure 2B**.

## 6 Therapeutic strategies targeting the neuro-immune-cardiovascular axis

### 6.1 Interventions modulating autonomic tone

#### 6.1.1 Device-based neuromodulation

Renal denervation (RDN) for treatment-resistant hypertension works partly by reducing renal and systemic neuro-driven immune activation. Carotid baroreceptor stimulation therapy for heart failure aims to reflexively reduce sympathetic and enhance vagal tone, improving cardiac function and suppressing inflammation.

Device-based neuromodulation represents a direct therapeutic strategy to correct autonomic imbalance, a core pathophysiological feature in cardiovascular diseases. Renal denervation (RDN), an ablation procedure targeting the renal sympathetic nerves, has been established as a treatment for resistant hypertension [established therapy, clinical use]. Its mechanism extends beyond simple blood pressure reduction; it partially works by diminishing the local renal and subsequent systemic neurogenic drive that activates immune pathways (66). By interrupting sympathetic efferent signals to the kidney, RDN reduces norepinephrine release, which in turn modulates adrenergic receptor signaling on immune cells and within the renal vasculature, thereby attenuating a pro-inflammatory state that contributes to vascular and renal injury (66). This aligns with the understanding that sympathetic hyperactivity promotes inflammation via catecholamine surges (108). For heart failure with reduced ejection fraction (HFrEF), carotid baroreflex activation therapy (BAT) has emerged as a promising intervention [investigational therapy, Phase III completed with neutral primary endpoint]. BAT involves electrical stimulation of the carotid sinus baroreceptors, which reflexively increases parasympathetic (vagal) outflow and decreases sympathetic nervous system activity (109). This restoration of autonomic balance is therapeutic; enhanced vagal tone activates the cholinergic anti-inflammatory pathway, while reduced sympathetic drive decreases catecholaminergic pro-inflammatory effects (110). Clinical trials, such as BeAT-HF, have demonstrated that BAT safely and sustainably improves patient-centered outcomes including quality of life, exercise capacity (6-minute walk distance), functional class, and reduces N-terminal pro-B-type natriuretic peptide levels in symptomatic HFrEF patients (111, 112). The therapy’s efficacy is linked to its ability to modulate the neuro-cardiac axis, improving left ventricular function and potentially reducing hospitalization rates, although its impact on hard cardiovascular mortality endpoints requires further long-term study (111, 113). These device-based approaches exemplify the translation of neuro-immune-cardiovascular axis principles into clinical practice by targeting the autonomic nervous system’s efferent output to achieve systemic anti-inflammatory and hemodynamic benefits.

#### 6.1.2 Pharmacological vagus nerve stimulation

Development of highly selective α7nAChR agonists (e.g., GTS-21) aims to mimic the cholinergic anti-inflammatory pathway for treating atherosclerosis, heart failure, and other diseases characterized by excessive inflammation, currently in preclinical and early clinical trial stages.

Pharmacological vagus nerve stimulation focuses on chemically activating the cholinergic anti-inflammatory pathway (CAP) by targeting the α7 nicotinic acetylcholine receptor (α7nAChR). This strategy seeks to replicate the anti-inflammatory effects of vagal efferent signaling without the need for invasive device implantation. The CAP is a critical neuro-immune mechanism where acetylcholine released from vagal efferents binds to α7nAChRs on immune cells, such as macrophages, inhibiting the release of pro-inflammatory cytokines like tumor necrosis factor-alpha (TNF-α) and interleukin-1β (IL-1β) (114). Selective α7nAChR agonists, such as GTS-21, are therefore under investigation as potential therapeutics for conditions driven by chronic inflammation [preclinical/exploratory, Phase II ongoing], including atherosclerosis and heart failure. Preclinical studies support this approach; for instance, in murine models of atherosclerosis, vagus nerve stimulation (VNS) has been shown to reprogram the neuro-immune-metabolic axis, suppressing inflammatory activation and reducing plaque burden (115). The anti-inflammatory effects of VNS, mediated through CAP activation, have also been associated with improved clinical outcomes in cardiovascular diseases like myocardial ischemia/reperfusion injury and heart failure in both animal and human studies (116). Pharmacological activation of this pathway offers a more targeted and potentially systemic approach compared to electrical VNS, which can have off-target effects due to the vagus nerve’s mixed fiber composition (117). Research into these agonists is advancing through preclinical and early clinical stages, exploring their efficacy in modulating specific inflammatory cascades, such as the NLRP3 inflammasome pathway, which is implicated in cardiac injury (85). By directly engaging this key neuro-immune node, α7nAChR agonists represent a promising drug-based strategy to dampen pathological inflammation within the neuro-immune-cardiovascular axis, offering a complementary or alternative option to device-based neuromodulation for a spectrum of inflammatory cardiovascular diseases.

### 6.2 Drugs targeting specific immune-neural interaction nodes

#### 6.2.1 Targeting splanchnic neuroimmunity

Research investigates selective modulation of splenic immune cell output via local splenic nerve ablation or pharmacological blockade of specific adrenergic signals within the spleen, without causing systemic sympathetic blockade, offering new avenues for atherosclerosis treatment.

Targeting the neuroimmune interface within the spleen represents a precision strategy to modulate systemic inflammation without inducing broad autonomic side effects. The spleen is a major reservoir and activation site for immune cells, and its function is regulated by sympathetic innervation. Splanchnic (splenic) nerve activity releases norepinephrine, which via adrenergic receptors on splenic immune cells, can influence their proliferation, egress, and cytokine production (118). Research aims to selectively disrupt this local neuroimmune communication to attenuate pro-inflammatory immune responses implicated in diseases like atherosclerosis. Preclinical studies in large animal models, such as pigs, have demonstrated that chronic, conscious splenic nerve neuromodulation via an implanted cuff electrode can significantly reduce systemic levels of pro-inflammatory cytokines like TNF-α during endotoxemia and promote a pro-resolving lipid mediator profile (119). This approach leverages the anatomical and functional similarities between porcine and human splenic innervation, providing a translational pathway (120). The goal is to achieve localized immunomodulation—for instance, by using pharmacological agents that block specific adrenergic receptor subtypes (e.g., β2-adrenoceptors) on splenic leukocytes or by performing minimally invasive splenic nerve ablation. This selectivity is crucial because systemic sympathetic blockade can have undesirable cardiovascular effects. Evidence suggests that such targeted intervention can reshape the immune landscape; for example, reducing the output of pro-inflammatory monocytes from the spleen could limit their recruitment to atherosclerotic plaques (121). Furthermore, the neuroimmune circuit involving the vagus nerve, brainstem, and splenic nerve (the inflammatory reflex) can be engaged via vagus nerve stimulation to ultimately modulate splenic immune responses, highlighting the spleen as a critical effector organ in the cholinergic anti-inflammatory pathway (110, 122). Therefore, therapies focused on the splenic neuroimmune axis offer a novel, organ-specific strategy to dampen vascular inflammation and alter the course of atherosclerosis by intervening at a key integrative node of the neuro-immune-cardiovascular axis.

#### 6.2.2 Cytokine-specific antagonists

Based on the pivotal role of IL-1β in the axis, canakinumab (an IL-1β monoclonal antibody) demonstrated efficacy in reducing atherosclerotic event risk in the CANTOS trial [proof-of-concept validated in Phase III trial], serving as a successful paradigm for translating neuro-immune-cardiovascular axis theory into clinical practice. Therapies targeting other axis-related factors (e.g., IL-6, TNF-α) are also under exploration.

Cytokine-specific antagonists represent a direct pharmacological attack on inflammatory mediators that are critically positioned within the neuro-immune-cardiovascular axis. The CANTOS trial with canakinumab, a monoclonal antibody against interleukin-1β (IL-1β), stands as a landmark demonstration that selectively inhibiting a single pro-inflammatory cytokine can significantly reduce recurrent cardiovascular events in patients with prior myocardial infarction and elevated high-sensitivity C-reactive protein, independent of lipid-lowering (121). This success validates the pathogenic role of inflammation, particularly the IL-1β-driven NLRP3 inflammasome pathway, in atherosclerosis progression and provides a clinical proof-of-concept for the neuro-immune-cardiovascular axis theory (85). IL-1β is a master regulator of inflammation whose production can be influenced by autonomic signals; sympathetic activation can promote its release, while vagal activity can suppress it via the cholinergic anti-inflammatory pathway (114). Beyond IL-1β, other cytokines integral to the axis are therapeutic targets. For instance, interleukin-6 (IL-6) has complex roles, with acute exercise-induced IL-6 release possessing anti-inflammatory properties, but chronic elevation is pro-atherogenic (123). Tocilizumab, an IL-6 receptor antagonist, is being explored in cardiovascular contexts. Similarly, tumor necrosis factor-alpha (TNF-α) is a key pro-inflammatory cytokine implicated in heart failure and atherosclerosis, and TNF-α inhibitors are used in autoimmune diseases, with ongoing research into their cardiovascular effects (116). The development of these biologic agents is grounded in the understanding that autonomic dysfunction (e.g., sympathetic overdrive) contributes to a systemic pro-inflammatory cytokine milieu, which in turn damages the vasculature and heart (14, 108). By directly neutralizing these cytokines, such therapies interrupt a downstream effector arm of the neuro-immune dialogue, offering a potent anti-inflammatory strategy. This approach complements neuromodulatory strategies by targeting the immune component of the axis, and its clinical success with canakinumab paves the way for further investigation into personalized anti-cytokine therapies for cardiovascular diseases. The key clinical trials that have evaluated therapeutic strategies targeting the neuro-immune-cardiovascular axis are summarized in **Table 2**. Beyond targeting individual cytokines, therapeutic strategies are increasingly focused on reshaping macrophage functional states—redirecting activation away from pathogenic pro-inflammatory trajectories (driven by chronic sympathetic overstimulation and NLRP3 inflammasome activation) towards homeostatic, inflammation-resolving, or reparative functional states. For example, α7nAChR agonists promote a regulatory macrophage phenotype characterized by IL-10 production and efferocytosis capacity, while β2-AR antagonists may attenuate sympathetic-induced pro-inflammatory macrophage differentiation. This nuanced approach recognizes that macrophages exist along a continuum of activation states and that therapeutic intervention should aim to modulate the overall activation landscape.

**Table 2:** Key clinical trials targeting the neural-immune-vascular axis.

Trial	Intervention	Population	Primary endpoint	Key finding
CANTOS	Canakinumab (anti-IL-1β monoclonal antibody)	Post-MI patients with hs-CRP ≥2 mg/L	MACE (non-fatal MI, stroke, CV death)	↓ MACE by 15% (HR 0.85, P = 0.021); validated inflammation as a therapeutic target in atherosclerosis (124)
BeAT-HF	Baroreflex activation therapy (BAT)	HFrEF (NYHA class III, LVEF ≤35%, NT-proBNP <1600 pg/mL)	Cardiovascular mortality and HF morbidity	Primary endpoint was neutral (rate ratio 0.94,P = 0.82); however, BAT significantly improved QoL, 6MHWD, and NYHA functional class (111)
SYMPLICITY HTN-3	Renal denervation (RDN)	Resistant hypertension	Office BP change at 6 months	Failed to meet primary efficacy endpoint; highlighted importance of patient selection and procedural standardization (125)
SPYRAL HTN-OFF MED	Renal denervation (RDN)	Resistant hypertension (off antihypertensive medications)	24-hour ambulatory BP change	Significant reduction in 24-hour ambulatory BP; re-established RDN efficacy with improved trial design (126)
INOVATE-HF	Vagus nerve stimulation (VNS)	Chronic HF, NYHA class III, LVEF ≤40%	Composite of all-cause death or first HF worsening event	Primary endpoint not met, trial terminated early for futility (127)

### 6.3 Lifestyle and multimodal interventions

#### 6.3.1 Axis-modulating effects of exercise training

Regular aerobic exercise is proven to enhance vagal tone, reduce sympathetic activity, and induce the release of anti-inflammatory myokines (e.g., acute IL-6 fluctuations have anti-inflammatory effects) and modulate immune cell function, serving as an effective non-pharmacological means of regulating the entire axis [recommended by current guidelines, clinical evidence supports efficacy].

Regular aerobic exercise is a powerful, non-pharmacological intervention that exerts comprehensive modulatory effects across the neural-immune-cardiovascular axis. Exercise training induces a favorable shift in autonomic balance, characterized by a sustained enhancement of parasympathetic (vagal) tone and a reduction in sympathetic nervous system activity (123). This autonomic remodeling is measurable through increased heart rate variability (HRV), particularly in high-frequency components, reflecting improved vagal modulation (128, 129). Concurrently, exercise drives immunometabolic reprogramming. Muscle contraction releases myokines, such as interleukin-6 (IL-6), which during acute exercise acts in an anti-inflammatory manner by stimulating the production of interleukin-10 (IL-10) and inhibiting TNF-α (123). This acute, transient IL-6 surge is a key mediator of exercise’s anti-inflammatory effects. Furthermore, exercise modulates immune cell function, for example, by promoting a less inflammatory phenotype in monocytes and macrophages and enhancing regulatory T cell (Treg) activity, partly through mechanisms involving short-chain fatty acids (123). These integrated effects—autonomic rebalancing and immune modulation—synergistically improve cardiovascular health. Exercise suppresses sympathetic overactivity, which is a driver of chronic inflammation and adverse cardiac remodeling (14). The enhanced vagal tone reinforces the cholinergic anti-inflammatory pathway, providing a neural brake on inflammation (108). Precision medicine approaches are now being applied to optimize exercise prescriptions, considering factors like individual genetic variability (e.g., ACE I/D genotype) to maximize these axis-modulating benefits (123). Therefore, structured exercise serves as a foundational lifestyle intervention that concurrently addresses the neural, immune, and vascular components of the axis, reducing systemic inflammation, improving endothelial function, and lowering cardiovascular risk.

#### 6.3.2 Mind-body interventions and stress management

Mindfulness meditation, yoga, and other behavioral interventions can reduce sympathetic nervous system activity and levels of inflammatory markers, regulating the neural-immune-cardiovascular axis from the “highest level,” and providing adjunctive value for primary and secondary prevention of cardiovascular disease.

**Figure 3:**
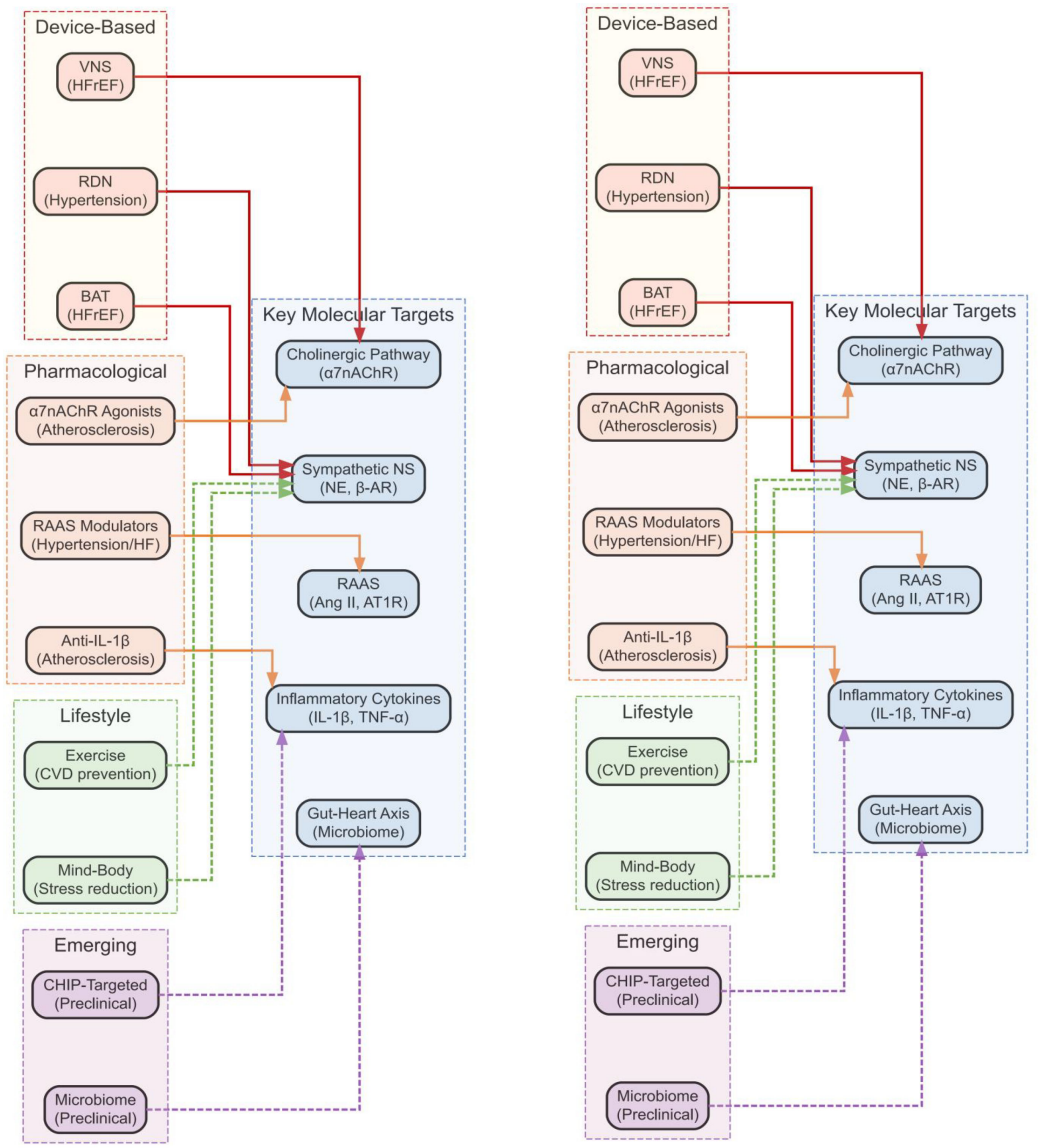
Therapeutic landscape of the neural-immune-vascular axis. Interventions are categorized by mechanism and development stage. Device-based strategies (red): RDN (hypertension), BAT and VNS (HFrEF), splenic nerve modulation (preclinical). Pharmacological (orange): anti-IL-1β (canakinumab, Phase III), α7nAChR agonists (GTS-21, Phase II), RAAS modulators (standard care). Lifestyle (green): exercise, mind-body practices (recommended). Emerging (purple): CHIP-targeted therapies, gut microbiome modulation (preclinical). Solid lines: established/clinical pathways; dashed lines: preclinical/exploratory. BAT, baroreflex activation therapy; CHIP, clonal hematopoiesis of indeterminate potential; GTS-21, selective α7nAChR agonist; HFrEF, heart failure with reduced ejection fraction; RAAS, renin-angiotensin-aldosterone system; RDN, renal denervation; VNS, vagus nerve stimulation.

Mind-body interventions such as mindfulness meditation, yoga, and controlled breathing techniques represent top-down strategies to regulate the neuro-immune-cardiovascular axis by modulating central nervous system processing and autonomic outflow. These practices effectively reduce psychological stress, a potent activator of the sympathetic nervous system and the hypothalamic-pituitary-adrenal (HPA) axis, both of which promote inflammation (43). For instance, yoga has been shown to favorably influence autonomic function, shifting the balance towards parasympathetic dominance, and to mitigate negative emotions, thereby attenuating the stress-induced vascular inflammation that contributes to non-communicable diseases (130). Specific techniques like resonance frequency breathing with heart rate variability biofeedback (HRV-BF) are designed to enhance parasympathetic tone and autonomic flexibility, leading to measurable increases in HRV parameters and improved cardiovascular adaptability (128, 129). Studies demonstrate that yoga practices, including Kriya Yoga and Anulom Vilom pranayama, can significantly reduce stress indices and enhance parasympathetic activity, while cooling pranayamas like Chandra Nadi are particularly effective for stress management (131). These behavioral interventions lower systemic inflammation by reducing the sympathetic drive that otherwise stimulates catecholamine release and pro-inflammatory cytokine production (14). Furthermore, they can improve baroreflex sensitivity and promote neural synchronization (129). By engaging higher cortical and limbic regions, mind-body practices modulate the “central command” of the autonomic nervous system, thereby exerting downstream effects on immune function and vascular health (132). Their ability to lower inflammatory markers and improve autonomic balance provides strong rationale for their incorporation as adjunctive tools in both primary and secondary cardiovascular disease prevention, offering a holistic approach to mitigating the detrimental impact of chronic stress on the neural-immune-cardiovascular axis. A comprehensive overview of therapeutic strategies targeting the neuro-immune-cardiovascular axis—categorized by intervention type and molecular target—is presented in **Figure 3**.

### 6.4 contradictory and negative evidence: lessons from failed or inconclusive trials

While the neuro-immune-vascular axis offers promising therapeutic targets, it is equally important to acknowledge the contradictory and negative evidence that has emerged from clinical trials. The neutral results of SYMPLICITY HTN-3 for renal denervation and INOVATE-HF for vagus nerve stimulation underscore the gap between mechanistic promise and clinical efficacy. In the anti-inflammatory domain, TNF-α blockade with etanercept and infliximab failed to improve outcomes in heart failure patients, with some studies even suggesting harm at higher doses—a cautionary tale for cytokine-targeted approaches. Similarly, broad anti-inflammatory strategies in heart failure have yielded mixed results: while the CANTOS trial demonstrated cardiovascular benefit in post-MI patients, the neutral results of the CIRT trial (low-dose methotrexate) and the modest effect size in heart failure populations highlight the need for patient selection and precision targeting. These negative and inconclusive trials are not failures but valuable learning opportunities. They suggest that: the neuro-immune-vascular axis is highly context-dependent, with efficacy varying by disease stage, patient phenotype, and intervention timing; broad immunosuppression is unlikely to succeed; and future trials must incorporate biomarker-based patient stratification to identify responders versus non-responders. A balanced discussion of both positive and negative evidence is essential for advancing the field beyond unbridled optimism toward precision neuromodulation and immunotherapy.

Throughout this section, therapeutic strategies are categorized according to the level of supporting evidence. Established therapies are those with regulatory approval or inclusion in clinical guidelines (e.g., RDN for hypertension, ACEI/ARB for heart failure). Investigational therapies have completed Phase II or III trials but lack definitive regulatory approval or have shown mixed results (e.g., BAT, VNS). Proof-of-concept validated strategies have positive Phase III trial results but are not yet broadly adopted (e.g., canakinumab for atherosclerosis). Preclinical/exploratory approaches have supportive animal or mechanistic data but lack clinical validation (e.g., α7nAChR agonists, CHIP-targeted therapies). Speculative strategies are based on mechanistic inference without direct human evidence (e.g., targeted microbiome modulation for cardiovascular indications). This classification is intended to provide readers with a transparent assessment of the current state of evidence for each therapeutic modality discussed.

## 7 Challenges and future prospects

### 7.1 Depth and complexity of mechanistic research

#### 7.1.1 Analysis of spatiotemporal specificity

The intricate and dynamic nature of the neural-immune-cardiovascular axis necessitates the application of advanced technologies to precisely map its interactions within specific spatiotemporal contexts of cardiovascular disease. Traditional bulk analyses often obscure the nuanced, real-time communication between nerve terminals, immune cells, and vascular cells within distinct microenvironments. Emerging single-cell and spatial transcriptomic approaches are critical for deconvoluting this cellular heterogeneity. For instance, in the context of atherosclerosis, specialized neuroimmune cardiovascular interfaces (NICIs) and artery tertiary lymphoid organs (ATLOs) form in the adventitia adjacent to plaques, acting as hubs that connect the plaque to the brain via multisynaptic projections and receive neural signals (133). To fully decipher these circuits, technologies like spatial transcriptomics are needed to define the molecular signatures of cells within these specific niches, such as the plaque shoulder or perivascular adipose tissue. Furthermore, understanding the real-time dynamics requires tools like optogenetics, which can be used to selectively stimulate or inhibit specific neural pathways (e.g., sympathetic or sensory fibers innervating blood vessels) to observe immediate effects on local immune cell behavior (e.g., macrophage polarization) and endothelial function (134). This approach is exemplified in cancer neuroscience, where the peripheral nervous system’s recruitment and innervation dynamically shape the tumor microenvironment by influencing immune and vascular cells (135). Similarly, in cerebral small vessel disease (CSVD), NG2-expressing glial cells dynamically interact with neurons, blood vessels, and other glia to regulate neurovascular integrity and immune signaling, processes that vary across disease stages and brain regions (136). Therefore, integrating single-cell resolution with spatial mapping and functional neural manipulation is paramount for constructing a high-fidelity, dynamic atlas of neuro-immune-cardiovascular crosstalk at critical disease sites.

#### 7.1.2 Integration of the gut-brain-heart axis

The influence of the gut microbiota extends far beyond the gastrointestinal tract, profoundly impacting systemic immunity and neural function, thereby positioning the gut as a key modulator within the broader neuro-immune-cardiovascular network relevant to cardiometabolic diseases. Future research must integrate the gut-brain axis into an expanded “neuro-immune-cardiovascular-gut” framework to understand its holistic role. The gut microbiota and its metabolites, transported via extracellular vesicles (mEVs) or circulation, can directly and indirectly influence cardiovascular health. For example, gut-derived mEVs can traverse intestinal and vascular barriers, carrying bioactive cargo that activates immune cells and influences neuroinflammation, a concept forming a “microbiota-EV-immune-neuro axis” relevant to conditions like Alzheimer’s disease, which shares vascular pathology with cardiovascular disorders (137). This mirrors the established “gut-liver axis,” where microbial metabolites regulate immune cell differentiation (e.g., Tregs) and cytokine production, with dysbiosis triggering immunological dysregulation through vascular, biliary, and neural pathways (138). In cardiovascular contexts, such gut-mediated immune modulation could directly affect vascular inflammation and atherosclerosis progression. Furthermore, neural pathways, such as the vagus nerve, provide a direct communication line between the gut and the cardiovascular system, influencing heart rate, inflammation, and vascular tone (134). The spleen serves as a crucial neuro-immune crossroads, with splenic innervation and immune cell dynamics (e.g., T lymphocytes) being implicated in blood pressure control, suggesting a potential point where gut-derived signals could integrate (139). Thus, a comprehensive model must account for how microbial metabolites (e.g., short-chain fatty acids), neural signals from the enteric and central nervous systems, and systemic immune activation collectively converge to influence vascular endothelial function, arterial stiffness, and the development of conditions like hypertension and coronary microvascular disease (140). This integrative perspective is essential for developing novel therapeutic strategies, such as probiotics or fecal microbiota transplantation, targeted at this axis for cardiometabolic disease prevention and management.

### 7.2 Precision and safety in therapeutic translation

#### 7.2.1 Personalized treatment strategies

The network-like complexity and individual variability of the neuro-immune-cardiovascular axis underscore the imperative for personalized medicine. Future therapeutic development must focus on identifying robust biomarkers to stratify patients who would most benefit from specific axis-targeted interventions. These biomarkers could encompass specific neurochemical profiles, immune cell subset signatures, or vascular reactivity patterns. For instance, in coronary microvascular disease (CMVD), profiling the activity of specific neuro-immune cardiovascular circuits and associated signaling pathways (e.g., NF-κB, STAT3) could identify patients with a predominantly inflammatory or dysautonomic etiology, guiding the use of anti-inflammatory agents like colchicine or specific neuromodulators (140). Similarly, in glioma, targeting the platelet-derived growth factor receptor alpha (PDGFRA), an oncogenic driver that orchestrates immune evasion and vascular abnormalities, requires biomarkers to identify tumors with PDGFRA-driven immunosuppression for vaccine-based therapies (141). In diabetic wound healing, the local expression level of neuropeptides like calcitonin gene-related peptide (CGRP) could serve as a biomarker to select patients for therapies designed to restore the “neuro-immune-cardiovascular” axis (142). The analysis of nerve density, proximity to the basement membrane, and perineural immune cell exclusion (e.g., CD8+ T cells) in cervical carcinogenesis provides spatially explicit metrics that could be used for risk stratification (143). Furthermore, minimally invasive biomarkers are emerging; for example, microbiota-derived extracellular vesicle (mEV) signatures in biofluids or cerebrospinal fluid (CSF) levels of proteins like neurofilament-light (NEFL) could reflect neurovascular pathology and treatment response (137, 144). Therefore, leveraging multi-omics approaches (genomics, transcriptomics, proteomics) to define patient-specific “axis signatures” will be crucial for moving from empirical treatments to precision-oriented interventions that modulate specific nodes of this interconnected network.

#### 7.2.2 Avoiding immunosuppression risks

Long-term therapeutic modulation of the immune component within the neuro-immune-cardiovascular axis necessitates a delicate balance between achieving anti-inflammatory efficacy and avoiding the pitfalls of generalized immunosuppression, such as increased infection or cancer risk. Consequently, developing strategies with enhanced specificity—such as tissue-targeted, conditionally activated, or context-dependent therapies—is a critical direction. Traditional broad immunosuppressants are unsuitable for chronic conditions like atherosclerosis or neurodegenerative diseases. Instead, approaches that locally reprogram immune cell function are preferred. For example, in Alzheimer’s disease pathology, Kv1.3 channel blockers alleviate neuroinflammation by specifically modulating microglial STAT1/3 signaling and type II interferon pathways, reducing plaque burden without causing widespread immunosuppression, as evidenced by increased synaptic and resilience proteins (144). Similarly, engineered mesenchymal stem cell-derived exosomes (MSC-Exos) can be designed to preferentially reprogram macrophage polarization in diabetic bone niches, alleviating inflammation and promoting angiogenesis without systemic immune suppression (145). The concept of “tumor-associated macrophage (TAM) reprogramming” in oncology uses pH/ROS-responsive nanoparticles or chimeric antigen receptor macrophages (CAR-M) to switch protumoral macrophages to an antitumoral phenotype specifically within the tumor microenvironment (146). In the context of cardiovascular disease, targeting neuroimmune hubs like artery tertiary lymphoid organs (ATLOs) in the adventitia could allow for localized immunomodulation without affecting systemic immunity (133). Furthermore, biomaterial-based delivery systems, such as glucose-responsive hydrogel microspheres for sustained release of CGRP in diabetic wounds, ensure the therapeutic agent acts primarily at the site of injury (142). These strategies highlight the shift from systemic immunosuppression to precise, spatially and temporally controlled immune modulation, thereby maximizing therapeutic benefit while minimizing off-target risks.

#### 7.2.3 Multi-target combination therapies

Given the inherently networked and redundant nature of the neuro-immune-cardiovascular axis, monotherapies targeting a single component may be insufficient or lead to compensatory mechanisms. Future treatment paradigms will likely favor rational, low-dose combination therapies that synergistically modulate neural, immune, and vascular pathways to enhance efficacy and reduce the side effects associated with high doses of any single agent. This approach leverages the interconnectedness of the axis for a more holistic intervention. For instance, in stroke, which involves a vascular-immune-neural network (VIN), combination cytoprotective strategies might simultaneously target vascular integrity (e.g., protecting the blood-brain barrier), dampening detrimental neuroinflammation, and supporting neural resilience (147). In glioma immunotherapy, PDGFRA-targeted vaccines are proposed to be combined with immune checkpoint inhibitors to overcome the profoundly immunosuppressive tumor microenvironment (141). For diabetic complications, a synergistic strategy is exemplified by CGRP-releasing microspheres that concurrently modulate macrophage function (immune), promote endothelial cell migration and tube formation (vascular), and restore local neuropeptide levels (neural), thereby achieving comprehensive healing (142). In coronary microvascular disease, combining a neuromodulator (to correct autonomic imbalance) with a low-dose anti-inflammatory agent (to address vascular inflammation) could be more effective than either alone (140). The field of acupuncture research provides a natural model of multi-target modulation, as it activates neurons, modulates immune responses, and influences vascular activity through a complex neuro-endocrine-immune network (148). Similarly, engineered neural constructs for tissue regeneration are designed to stimulate osteogenesis, angiogenesis, and neuromuscular junction formation simultaneously, reflecting a multi-system approach (149). Therefore, designing combination therapies requires a deep understanding of the key signaling nodes and feedback loops within the axis to identify synergistic, low-dose drug pairs or multi-functional biologics that collectively restore homeostasis with an improved safety profile.

## 8 Conclusion

The neuro-immune-cardiovascular axis represents a paradigm shift in our understanding of cardiovascular disease, moving beyond traditional siloed approaches to embrace a holistic, systems-level perspective. This integrative framework compellingly demonstrates that the nervous and immune systems are not merely passive responders or bystanders in vascular pathology but are active, indispensable drivers and dynamic regulators. The journey from chronic sympathetic overactivation, which fuels a pro-inflammatory and pro-remodeling milieu, to the suppression of the protective cholinergic anti-inflammatory pathway, underscores a fundamental dysregulation of homeostatic communication. This axis operates across scales—from the intimate, synapse-like interactions at neuro-immune junctions within the vascular wall to the systemic cascades of inflammatory storms seen in conditions like myocarditis or severe atherosclerosis. This complexity, while daunting, reveals a rich tapestry of potential therapeutic targets.

From an expert standpoint, the development of this conceptual axis marks a critical maturation in cardiovascular medicine. It forces a reconciliation between historically distinct fields—cardiology, neurology, and immunology—demanding that we view the heart and vessels not as isolated pumps and pipes but as organs deeply embedded within and responsive to the body’s neural and defense networks. The impact is twofold: diagnostically, it provides a mechanistic explanation for the established links between chronic stress, inflammation, and adverse cardiovascular outcomes; therapeutically, it opens entirely new avenues for intervention that were previously inconceivable within a purely hemodynamic or metabolic model.

The translation of this knowledge into clinical practice is already underway, showcasing a spectrum of innovative strategies. On one end, device-based neuromodulation therapies, such as renal denervation for resistant hypertension or vagus nerve stimulation trials in heart failure, aim to rebalance autonomic input directly. On the other end, biologic agents targeting specific cytokines or immune cell receptors, born from rheumatology and oncology, are being repurposed to quell vascular inflammation, as seen with IL-1β inhibition in atherosclerosis. This diversification of approach is a direct result of embracing the axis’s multifaceted nature.

Balancing the different research perspectives and findings within this field is paramount. Reductionist studies identifying specific neurotransmitters, immune cell subtypes, or signaling molecules are essential for pinpointing precise drug targets. However, these must be integrated with systems biology approaches that model the network’s emergent properties and temporal dynamics. For instance, while inhibiting a specific pro-inflammatory cytokine may be beneficial in a chronic setting, the same inhibition could be detrimental during an acute infection or wound healing. Similarly, broad autonomic suppression might lower blood pressure but could impair physiological stress responses. The challenge lies in achieving precision—intervening in the right pathway, at the right time, in the right patient.

The major hurdles ahead are significant but surmountable. First, we must move from a static to a dynamic understanding, mapping the spatial and temporal evolution of neuro-immune-cardiovascular crosstalk across different disease stages (initiation, progression, acute decompensation). Advanced imaging, single-cell omics, and real-time biosensors will be crucial here. Second, therapeutic precision and safety demand better patient stratification. Biomarkers that reflect an individual’s “axis phenotype”—such as their autonomic tone, immune cell repertoire, or vascular reactivity—are needed to predict who will benefit from neural modulation versus immunomodulation. Finally, the long-term effects of modulating this fundamental axis require careful scrutiny in large-scale trials.

In conclusion, the neuro-immune-cardiovascular axis is more than a novel concept; it is a necessary lens through which the future of cardiovascular medicine must be viewed. Its development underscores a transition from organ-centric to network-centric thinking. The path forward hinges on deep, interdisciplinary collaboration that bridges basic neuroscience, immunology, vascular biology, and clinical trial design. By continuing to unravel the intricate dialogues within this axis, we can transform these insights into the next generation of targeted, effective, and safe therapies, ultimately translating a profound understanding of interconnectedness into tangible improvements in cardiovascular patient outcomes.
